# Bicarbonate in AKI and acidemia to reduce mortality and need for kidney replacement therapy

**DOI:** 10.1093/ckj/sfaf398

**Published:** 2025-12-18

**Authors:** Rumen Filev, Turgay Saritas

**Affiliations:** Department of Nephrology, UMHAT Saint Anna, Sofia, Bulgaria; Internal Medicine Department, Medical University Sofia, Bulgaria; Department of Nephrology, University Hospital RWTH Aachen, Aachen, Germany; Department of Internal Medicine, Nephrology and Transplantation, Erasmus Medical Center Rotterdam, The Netherlands

Severe metabolic acidemia (arterial pH ≤7.20) in the setting of acute kidney injury (AKI) remains a major clinical challenge in the intensive care unit (ICU) [1]. Acidemia may impair cardiac contractility, worsen kidney perfusion and contribute to multi-organ dysfunction [2]. The BICARICU-2 (Sodium Bicarbonate for the Treatment of Severe Metabolic Acidosis With Moderate or Severe Acute Kidney Injury in ICU) investigators conducted a pragmatic multicenter, open-label, randomized trial to determine whether intravenous sodium bicarbonate (target pH ≥7.30), compared with usual care without bicarbonate, would improve outcomes in adults with severe acidemia and KDIGO stage 2–3 AKI [1, 3].

The study enrolled 640 patients from 43 French ICUs between October 2019 and December 2023. A total of 627 patients (314 assigned to bicarbonate, 313 to control) were included in the intention-to-treat analysis. Baseline characteristics were well balanced: median age 67 years, predominantly septic shock, median arterial pH around 7.15 and serum bicarbonate 12–13 mEq/L, and a high proportion required vasopressor support (∼80%) or mechanical ventilation (∼75%).

The key findings were as follows (Fig. 1):

• Primary outcome: 90-day all-cause mortality was similar between groups—195/314 (62.1%) in the bicarbonate arm vs 193/313 (61.7%) in the control group [absolute difference 0.4%; 95% confidence interval (CI) –7.2% to +8.0%; *P* = .91].
• Secondary outcome initiation of kidney replacement therapy (KRT): a significant reduction in KRT use by Day 28 was observed in the bicarbonate group (35% vs 50%; absolute difference –15.5%; 95% CI –23.1% to –7.8%).
• Timing of KRT: median time to KRT initiation was delayed with bicarbonate (30.9 h vs 15.5 h).
• Other outcomes: ICU and hospital length of stay, days free of mechanical ventilation or vasopressors, major adverse kidney events at Day 90 and adverse-event rates were similar in the two groups.

**Figure 1:**
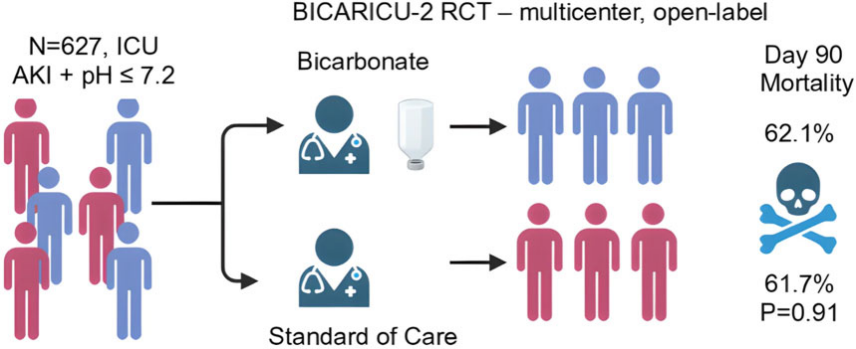
Design and outcomes of the BICARICU-2 randomized controlled trial.

## INTERPRETATION AND CLINICAL IMPLICATIONS

The trial demonstrated a clearly neutral primary outcome: sodium bicarbonate therapy did not reduce 90-day mortality in critically ill patients with severe acidemia and moderate-to-severe AKI. From a nephrology perspective, this suggests that routine bicarbonate infusion for the purpose of improving survival in this population is not supported [1, 4].

However, the marked reduction in KRT initiation represents an important secondary finding. This suggests bicarbonate may delay or decrease the need for dialysis, potentially by alleviating acidemia-related triggers for initiating KRT. Because dialysis is resource intensive and associated with complications, any intervention that safely reduces its use may have clinical relevance [4].

Several considerations temper the interpretation of these results. The study was powered to detect a relatively large absolute reduction in mortality, based on earlier estimates of very high mortality in this population [2]. The observed mortality was substantially lower, increasing the possibility that smaller but meaningful survival effects could not be detected. Additionally, open label design may have influenced non-standardized indication for KRT. Patients receiving intravenous sodium bicarbonate received ∼750 mL more fluid in the first 48 h than controls, and whether this affected resuscitation or decisions to start KRT in moderate-to-severe AKI is also unclear.

Overall, for patients with severe acidemia and AKI, sodium bicarbonate infusion appears safe and may reduce or delay dialysis initiation but should not be considered a mortality-improving strategy. However, it remains unclear whether the lower KRT use with bicarbonate reflects a genuine reduction in AKI progression or mainly a change in clinical thresholds and timing for dialysis, given that acidosis is an important trigger and pharmacologic pH correction may simply keep patients from reaching that point. Even if it does not halt AKI progression, bicarbonate may still be beneficial by limiting exposure to KRT-related risks and their potential downstream harms.
